# Birefringence in Injection-Molded Cyclic Olefin Copolymer Substrates and Its Impact on Integrated Photonic Structures

**DOI:** 10.3390/polym16020168

**Published:** 2024-01-05

**Authors:** Stefan Kefer, Tobias Limbach, Natalie Pape, Kathrin Klamt, Bernhard Schmauss, Ralf Hellmann

**Affiliations:** 1 Applied Laser and Photonics Group, Aschaffenburg University of Applied Sciences, Wuerzburger Strasse 45, 63743 Aschaffenburg, Germanyralf.hellmann@th-ab.de (R.H.); 2 Plastic Centre Leipzig, Erich-Zeigner-Allee 44, 04229 Leipzig, Germany; klamt@kuz-leipzig.de; 3 Institute of Microwaves and Photonics, University of Erlangen-Nuremberg, Cauerstrasse 9, 91058 Erlangen, Germany; bernhard.schmauss@fau.de

**Keywords:** cyclic olefin copolymer, birefringence, integrated photonics, Bragg grating, injection molding

## Abstract

This contribution quantifies the birefringence within injection-molded cyclic olefin copolymer plates and discusses its impact on the mechanical properties of the plates. It also focuses on the impact of birefringence on integrated waveguides and Bragg gratings and provides fabrication guidelines for such structures. The anisotropy in all three dimensions of the workpiece is examined by means of polarimetry and a prism coupler. It is found that the birefringence is inhomogenously distributed within the workpieces, whereas the maximum birefringence not only varies locally, but also depends on the observation direction. Overall, a maximum birefringence of 10 × 10^−4^ is found at the plate’s surface near the injection gate. The anisotropy then reduces exponentially towards the center of the workpiece and saturates at 1.8 × 10^−4^, in a depth of 0.4 mm. Thus, the birefringence strongly affects near-surface photonic structures. It is found that, depending on their orientation and the local birefringence of the substrate, waveguides and Bragg gratings fabricated with comparable parameters behave completely differently in terms of polarization-dependent optical attenuation, cross-sectional intensity distribution and Bragg reflection signal. For example, the support of the TM mode can vary between total loss and an optical attenuation of 0.9 dB × cm^−1^. In consequence, this study underlines the importance of quantifying the birefringent state of an injection-molded cyclic olefin copolymer workpiece if it is supposed to serve as a substrate for integrated photonic structures. The study furthermore demonstrates that birefringence effects can be omitted by burying the photonic structures deeper into the volume of the thermoplastic.

## 1. Introduction

Integrated photonics based on planar polymer platforms is still a rapidly growing research field. In general, polymer-based photonic devices offer some intrinsic advantages over silica- and semiconductor-based platforms, such as their inherent photosensitivity, a low Young’s modulus and thus outstanding flexibility as well as comparably straightforward handling and machinability [1,2,3,4]. However, some standard polymers suffer from drawbacks, for example a limited capability to withstand elevated temperatures as well as, in the case of photonic sensing devices, an unwanted cross-sensitivity towards relative humidity fluctuations [5]. This can be overcome by employing cyclic olefin copolymer (COC) substrates, as this high-grade thermoplastic provides glass transition temperatures up to 250 °C as well as a vastly reduced water absorption down to 0.01% [6,7,8]. Based on these advantages, a multitude of photonic devices based on planar COC substrates have been developed throughout recent years. The most prominent examples include temperature sensors capable of evaluating temperatures up to 160 °C [9], multi-dimensional strain or shape sensors [10], electro-optic and optofluidic devices [11,12], hypersensitive hydrogen detectors [13], refractive index sensors [14] and lab-on-a-chip devices [15].

Most of these platforms are based on near-surface waveguides (WGs) and Bragg gratings (BGs). The BG acts as a selective reflector, whereas its reflection properties are susceptible to environmental influences. Mainly its wavelength of main reflection, which is also commonly denoted as Bragg wavelength, is then evaluated for sensing purposes [16]. These platforms are fabricated by means of a sophisticated single-writing-step procedure, which allows simultaneous generation of a near-surface WG comprising a BG [17]. With this process, BGs exhibiting a reflectivity of up to 98% and a spectral full width at half maximum (FWHM) of down to 350 pm can be manufactured. With an attenuation of 1.2 dB × cm^−1^, around wavelengths of 1550 nm, the respective waveguides are acceptable for on-chip waveguiding purposes [18]. Alternatively, novel femtosecond laser-based direct-writing approaches can be employed to enable fabrication of photonic structures that are buried deeper in the volume of the planar COC substrate. Such devices exhibit reflectivities of up to 95%, an FWHM of 288 pm and a waveguide attenuation of 3.2 dB × cm^−1^ in the C band [19].

Another advantage of planar COC-based photonic devices is the possibility to employ injection-molded substrates. This allows for cost-efficient manufacturing of the polymer platforms, especially in large quantities [20]. However, while the COC wrought material is intrinsically amorphous [21], injection-molding processes are well known for introducing anisotropies into a workpiece [22]. The root cause for this can be found in the flow dynamics during the filling of the mold and during the subsequent cooling step. This leads to a local preferred orientation direction of the polymer macromolecules, or polymer chains [23,24,25]. The resulting birefringence effects are also observable in injection-molded planar COC specimens [26]. Therefore, this study investigates and discusses the birefringence of injection-molded COC plates. Besides a three-dimensional analysis of the inhomogeneous birefringence distribution within the workpiece, the study evaluates mechanical analysis approaches for the determination of anisotropic effects. However, the article mainly focuses on the impact of the birefringence on integrated photonic waveguides and Bragg gratings.

## 2. Materials and Methods

### 2.1. Injection-Molded Cyclic Olefin Copolymer and Sample Preparation

**Figure 1:**
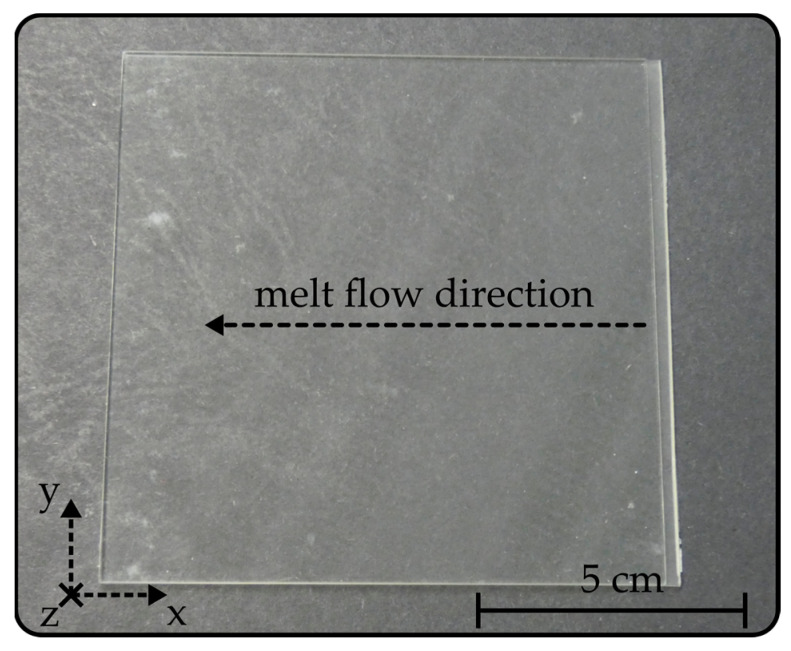
Exemplary photography of an injection-molded COC plate.

COC plates with a quadratic footprint of 100 × 100 mm² and a thickness of 1.5 mm were manufactured with an injection-molding machine (DEMAG ergotech 100/420-310s, Sumitomo (SHI) Demag Plastics Machinery, Schwaig, Germany) from the thermoplastic wrought material (TOPAS 6017S-04, TOPAS Advanced Polymers, Raunheim, Germany). The production of the plates was carried out at a melt temperature of 280 °C and an average mold temperature of 150 °C. The pellets were plasticized at a screw speed of 150 rpm and a back pressure of 8 MPa. Mold filling via a cold runner system with a film gate, in a time frame of 0.9 s, resulted in an injection pressure of 71.3 MPa. In relation to this, the holding pressure was graded over a time frame of 10 s. The cooling time in the process was 25 s and the overall cycle time amounted to 48 s. The final COC plates exhibited a high-gloss surface with a mean surface roughness of 10 nm, as confirmed by white-light interferometry (ContourGT, Bruker, Billerica, MA, USA). An exemplary image of an injection-molded COC plate is shown in Figure 1. Smaller samples with application-oriented geometries were cut from the COC plate by means of a micro mill (CNC Mini-Mill/4, Minitech Machinery, Norcross, GA, USA). Based on the employed parameters, a mean area surface roughness of 200 nm can be reached with circumferential milling processes [14].

### 2.2. Birefringence Characterization

**Figure 2:**
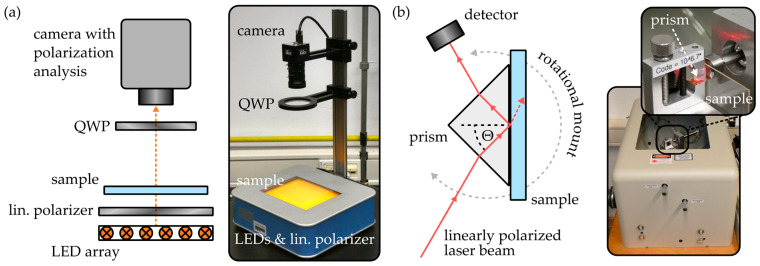
Working principle and images of the employed (**a**) real-time polarimeter and (**b**) prism coupler.

A polarimeter (StrainScope Flex, ilis, Erlangen, Germany) was used to determine the two-dimensional birefringence distribution within the injection-molded COC samples. The device, which operates on the Sénarmont compensation method [27], comprises a light-emitting diode (LED) array, a linear polarizer, a quarter wave plate (QWP) and a camera featuring integrated polarization analysis, as illustrated in Figure 2a.

The polarimeter is capable of quantifying the phase shift, or retardance φ, between the respective polarization components oriented parallel to the birefringent sample’s fast axis or slow axis if they are rotated in a 45° angle with respect to the linear polarizer’s transmission axis. Additional determination of the sample thickness h then enables calculation of the COC plate’s absolute, two-dimensional birefringence Δn according to

(1) $$\Delta n=\frac{\varphi }{h}.$$

Based on the LED emission wavelength of 594 nm, the maximum retardance value that can be quantified is specified as 280 nm. However, subsequent software-based phase unwrapping enables the measurement of larger retardance values. Based on the employed camera objective, samples with lateral extensions up to 152 × 114 mm^2^ can be evaluated with spatial resolutions down to 12 µm.

In order to determine the surface refractive index and birefringence of the COC samples, a prism coupler (2010/M, Metricon Corporation, Pennington, NJ, USA) was employed. As outlined in Figure 2b, the device exploits the total internal reflection principle to determine the refractive index of a thick, bulk material. Therefore, the sample is brought into contact with a high-index prism (n = 1.93429 at a wavelength of 1548 nm and a temperature of 25 °C) and a linearly polarized laser beam is guided through the prism onto the prism-to-sample interface. For incidence angles Θ, satisfying the total internal reflection condition

(2) $$\Theta \geq \arcsin\left(\frac{n_{S}}{n_{P}}\right),$$

the laser beam is reflected at the prism-to-sample interface and thus redirected onto the detector. If the total internal reflection condition is not fulfilled, the laser beam couples from the prism into the bulk sample, which leads to immediate signal loss. Consequently, by mounting the configuration on a rotational platform and thus varying the incidence angle, the sample’s refractive index can be found with a specified resolution of 2 × 10^−4^. Since the prism coupler is equipped with multiple light sources emitting at wavelengths of 637 nm, 830 nm, 1310 nm and 1548 nm, the results are comparable with those obtained with the polarimeter, as measurements can be conducted at a comparable wavelength. Moreover, it is also possible to approximate a sample’s dispersion curve with this setup. Furthermore, the polarization direction of the laser beam can be oriented either parallelly or orthogonally with respect to the substrate surface. Thus, the device also enables quantification of the local surface birefringence of an injection-molded COC specimen. All birefringence values acquired with the prism coupler and stated in the outline of this work represent the average birefringence at all four wavelengths.

### 2.3. Mechanical Characterization

**Figure 3:**
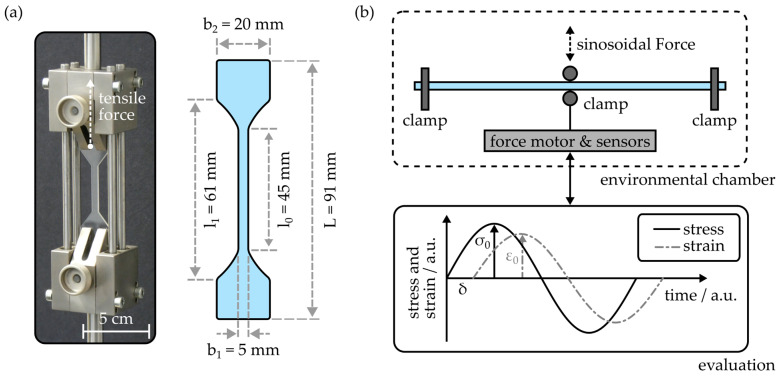
(**a**) Setup and specimen geometry for the determination of the tensile properties of injection-molded COC. (**b**) Working principle of the dynamic mechanical thermal analysis setup employed to examine the temperature-dependent flexural properties of the COC samples.

The static tensile properties of injection-molded COCs were examined by means of a tensile testing apparatus (EZ-LX, Shimadzu, Duisburg, Germany). Figure 3a depicts a photograph of the tensile test setup as well as a drawing of the tensile test sample design.

In principle, the sample design is based on the recommendations comprised in the ISO 527-2 standard [28]. However, to provide comparability with the conventional substrate extensions employed for the fabrication of COC-based integrated photonic devices, the specimen design was adapted. The respective shape dimensions are also provided in Figure 3a. During an experimental cycle, the sample was elongated up to its breaking point with a constant speed of 1 mm × min^−1^. Based on the residual stress σ, which can be calculated from the applied tensile force F and the sample dimensions via

(3) $$\sigma =\frac{F}{A}=\frac{F}{b_{1}h},$$

the tensile modulus E_tens_ of the injection-molded COC can be determined with

(4) $$E_{tens}=\frac{\sigma }{\varepsilon }=\frac{F\times l_{0}}{b_{1}h\times \Delta l}.$$

Here, ε represents the strain experienced by the tensile test sample, which is defined as the ratio of length change Δl over the initial length l_0_. Furthermore, the experiment provides information on the respective tensile stress and strain values at the specimen’s breaking point.

To examine the temperature-dependent flexural properties of the injection-molded COC, a dynamic mechanical thermal analysis (DMTA) setup (DMA 800, PerkinElmer, Inc., Waltham, MA, USA) was used. As outlined in Figure 3b, the device is employed in a three-point bending configuration, where a sinusoidal load is introduced to the central region of the specimen. For all DMTA experiments conducted in the outline of this study, slab-shaped specimens with a length of 50 mm, a width of 3 mm and a thickness of 1.5 mm were used. The distance between the clamps was 40 mm, while the sinusoidal force was applied with a frequency of 1 Hz and the maximum displacement d_0_ was set to 50 µm. Evaluation of the applied maximum stress $\sigma_{0}$, the resulting strain $\varepsilon_{0}$ and the phase shift δ yields the storage modulus E′ and the loss modulus E″ according to

(5) $$E^{\prime }=\frac{\sigma_{0}}{\varepsilon_{0}}\cos\delta$$

and

(6) $$E^{\prime \prime }=\frac{\sigma_{0}}{\varepsilon_{0}}\sin\delta ,$$

respectively. Based on storage and loss modulus, the absolute of the complex modulus |E*| is therefore given as

(7) $$\left|E*\right|=\sqrt{{E^{\prime }}^{2}+{E^{\prime \prime }}^{2}}=\frac{\sigma_{0}}{\varepsilon_{0}}.$$

Besides this property, which is commonly employed to quantify the flexural stiffness of a viscoelastic material, the damping factor

(8) $$\tan\delta =\frac{E^{\prime }}{E^{\prime \prime }}$$

can be used to identify the glass transition temperature [29,30,31]. For this, the DMTA setup is equipped with an environmental chamber, which enables exposition of the COC samples to temperatures ranging from 15 °C to 180 °C during the flexural experiments.

### 2.4. Integrated Photonic Structures

#### 2.4.1. Fabrication and Working Principle

**Figure 4:**
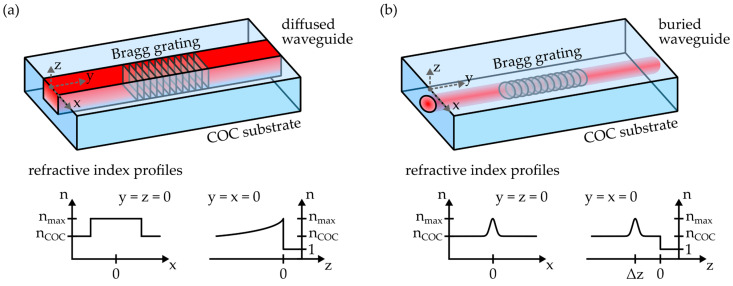
Schematics and refractive index profiles of (**a**) diffused waveguides, fabricated via the single-writing-step method, and (**b**) buried waveguides, manufactured via femtosecond laser-based direct writing. Both waveguide types can comprise Bragg grating structures.

In the outline of this study, two different fabrication methods were employed to generate integrated photonic structures within the injection-molded, planar COC substrates. The single-writing-step (SWS) method, a sophisticated mask exposure process, is based on covering the COC specimen with a phase mask and an amplitude mask. Subsequently, the stack is irradiated with ultraviolet laser radiation [17]. As shown in Figure 4a, this leads to the formation of a photonic waveguide and a Bragg grating (BG) structure directly underneath the substrate surface. The waveguide exhibits an asymmetric refractive index profile, with a step-index transition alongside the x-axis and a gradient-index depth profile parallel to the z-axis.

The waveguiding properties of the waveguide, as well as the reflection signal properties of the BG, are thereby mainly dependent on the employed fabrication parameters, i.e., amplitude mask width, phase mask periodicity as well as BG length and the applied laser fluence [18]. Due to its refractive index profile, this waveguide type is often referred to as a diffused waveguide [32]. All such photonic structures examined in this study were generated in COC substrates with a length of 20 mm and a width of 10 mm. The samples were irradiated by means of a KrF excimer laser (BraggStar Industrial, Coherent Europe B.V., Utrecht, The Netherlands), which emits ultraviolet light with a wavelength of 248 nm. The radiation propagates through an amplitude mask with a width of 27 µm and a phase mask with a periodicity of 1034 nm before it interacts with the polymer substrate. The overall fluence was 100 J × cm^−2^ with a repetition rate of 200 Hz. Afterwards, the photonic platforms were thermally treated at a temperature of 130 °C for 2 h. In comparison to the refractive index of the pristine COC n_COC_, the process resulted in a maximum positive refractive index modification n_max_ of 1 × 10^−3^ at the substrate surface [33].

Alternatively, femtosecond lasers can be used to fabricate buried waveguides and Bragg gratings in planar COCs. In contrast to diffused waveguides generated with the SWS method, these photonic structures exhibit a rotational-symmetric refractive index profile with a Gaussian cross-section, as depicted in Figure 4b. Furthermore, the writing depth can be deliberately chosen. However, in order to omit repeatability issues, all femtosecond laser-based structures in this work were located at least 50 µm underneath the substrate surface. The respective fabrication parameters for waveguides and Bragg gratings are summarized in Table 1.

After irradiation, the samples were also thermally treated at a temperature of 130 °C for a duration of 2 h. This resulted in a maximum refractive index modification of 4 × 10^−4^. Please note that an in-depth discussion of the process and the resulting photonic structures is provided by the authors in [19].

#### 2.4.2. Analysis of Integrated Waveguides and Bragg Gratings

**Figure 5:**
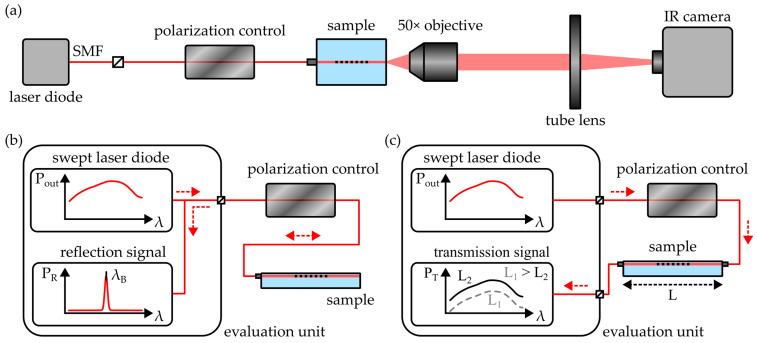
Schematics of the employed experimental setups for (**a**) near-field analysis, (**b**) quantification of the Bragg reflection signal and (**c**) determination of the waveguide attenuation by means of the cut-back method. All setups feature a polarization control unit for the distinct excitation of the waveguide’s TE or TM mode. Red, dashed arrows indicate the propagation direction of the radiation.

Multiple experimental setups were used to examine the performance of the integrated photonic structures. The mode-field distribution of the waveguides was evaluated by means of the near-field analysis microscope depicted in Figure 5a.

A pigtailed laser diode (LU1550M150-1A06F30H, Lumics, Berlin, Germany) emits linearly polarized radiation with a wavelength of 1550 nm. Subsequent in-line polarization control enables the adaptation of the polarization axis in order to provide explicit excitation of the integrated waveguide’s polarization modes, i.e., the TE and the TM mode, which oscillate parallel or perpendicular to the substrate surface, respectively. Afterwards, light is butt-coupled into the waveguide with a fiber-optic pigtail. After exiting the sample, the radiation is imaged onto the detector of an infrared camera (1460–1600 nm Near-Infrared Camera, Edmund Optics, Barrington, NJ, USA) by a 50× objective (M Plan Apo NIR HR 50×, Mitutoyo, Neuss, Germany) and a tube lens (TTL200-S8, Thorlabs, Bergkirchen, Germany). During subsequent evaluation, a near-field image is generated from 50 recorded frames. Furthermore, the raw data is processed with camera sensor linearization and dark frame correction algorithms.

The integrated Bragg grating structures act as a selective reflector, whereas their wavelength of main reflection, or Bragg wavelength λ_B_, is correlated with the grating’s effective refractive index n_eff_ and the grating period Λ by the Bragg relation

(9) $$m\lambda_{B}=2n_{eff}\Lambda .$$

Here, the integer m represents the order of the grating. The reflection signal of the BGs fabricated in this study were evaluated with a commercial-grade interrogation unit (Hyperion si155, Luna, Roanoke, VA, USA). It employs a swept laser diode to emit light with wavelengths ranging from 1460 to 1620 nm. Radiation reflected by the Bragg grating is detected and the wavelength of main reflection can be determined with a resolution of 1 pm at measurement frequencies of up to 1 kHz. A schematic of the setup is shown in Figure 5b. The setup was also equipped with an optional polarization control unit, which allows distinct excitation of a waveguide’s polarization modes. In the case of a birefringent waveguide, this leads to distinct reflection peaks with distinct Bragg wavelengths for each polarization mode. On the assumption that the Bragg wavelength difference λ_B,TE_ − λ_B,TM_ is solely based on the waveguide’s birefringence Δn, the relation of both properties is obtained from Equation (9) as

(10) $$\Delta n=\frac{m}{2\Lambda }\left(\lambda_{B,TE}-\lambda_{B,TM}\right)=\frac{m}{2\Lambda }∆\lambda_{B}.$$

In order to determine the optical attenuation of the integrated waveguides, a cut-back method was used. Therefore, the COC-based photonic platform was also connected to the interrogation device. However, instead of observing the Bragg reflection, the transmission signal was evaluated, as outlined in Figure 5c. Please note that while the examined devices comprise Bragg grating structures to enable straightforward coupling of the fiber-optic pigtails, the BGs do not interfere with this measurement, as spectral regions far from the Bragg reflection were analyzed. During the experiment, the length L of the examined waveguide was incrementally reduced in 0.5 mm steps and the transmission signal amplitude was reevaluated after each length reduction. This way, by analysis of the change in transmitted optical power ΔP_T_, in correlation with the length variation ΔL, the waveguide’s optical loss α is determined as

(11) $$\alpha =\frac{\Delta P_{T}}{\Delta L}.$$

For all experiments, light was butt-coupled into the integrated waveguides by means of a fiber-optic pigtail. Moreover, a UV-curable adhesive (NOA78, Norland Optical Adhesives, Jamesburg, NJ, USA) was used to establish a durable connection between the pigtail and COC substrate. The adhesive additionally serves a refractive index matching liquid to suppress any unwanted Fabry-Pérot interferences at the fiber-to-polymer interface.

## 3. Results and Discussion

### 3.1. Analysis of Three-Dimensional Birefringence

**Figure 6:**
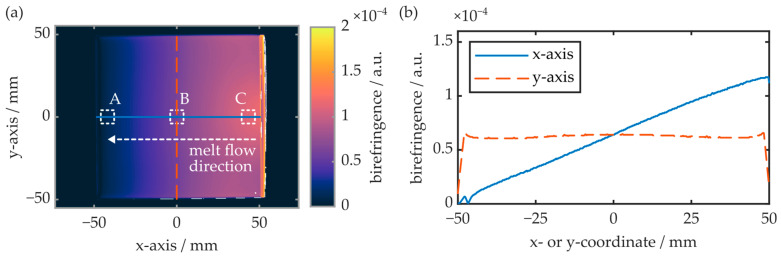
(**a**) False-color representation of an injection-molded COC plate’s birefringence in the xy-plane. A, B and C represent the respective areas for comparison measurements obtained via the prism coupler and subsequent polarimetry measurements in the xz- as well as the yz-plane. (**b**) Birefringence along the sample’s x- and y-axis.

The birefringence of the injection-molded COC plates was examined with the polarimeter. First, as shown in Figure 6, the birefringence parallel to the specimen’s xy-plane was evaluated.

**Figure 7:**
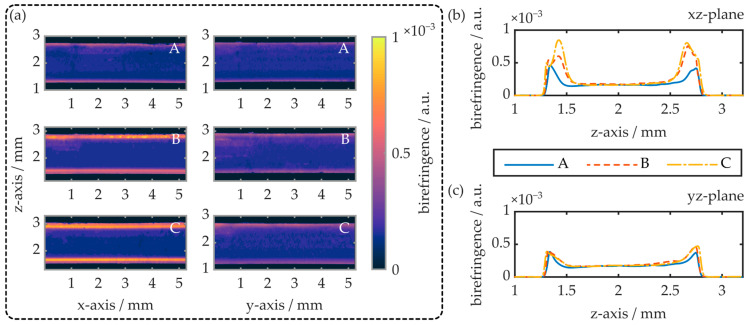
(**a**) Exemplary false-color representations of the birefringence in the xz- as well as the yz-plane of the injection-molded COC plates. (**b**,**c**) show the respective z-axis birefringence profiles. The samples were obtained from regions A, B and C of an injection-molded COC specimen (see Figure 6).

The experiment reveals that, on the x-axis and thus parallel to the melt flow direction, the COC plate exhibits maximum birefringence at the injection gate. With increasing distance from the gate, the birefringence decreases linearly and approaches zero at the COC plate’s opposite end. Parallel to the y-axis, and thus orthogonal to the melt flow direction, the birefringence is constant, whereas the absolute value is again determined by the distance between the measurement region and gate. The local surface birefringence at the positions A, B and C (see Figure 6a) is also determined by means of the prism coupler. While the surface birefringence at positions A and B is below the device’s theoretical measurement resolution (≤2 × 10^−4^), a surface birefringence of 5 × 10^−4^ is observed at position C. This value differs significantly from the birefringence observed via polarimetry, which amounts to approximately 1.2 × 10^−4^ at this position. This discrepancy can be explained by an inhomogeneous birefringence distribution throughout the COC plate’s z-axis. To examine this, slab-shaped samples with a length of 10 mm and a width of 0.2 mm were cut from the 1.5 mm thick COC plate. Again, the samples were taken from regions A, B and C, whereas their smallest extension (width) was oriented parallelly to the measurement plane. Thus, depending on the specimen orientation, the birefringence in the xz- as well as in the yz-plane was evaluated. A summary of the obtained results is provided in Figure 7.

The polarimetry results reveal an inhomogeneous birefringence distribution within injection-molded COC plates. In all cases, the specimens exhibit maximum birefringence at and just underneath both of the COC plate surfaces. Thereby, the maximum birefringence value depends on the specimen position as well as the measurement direction. Parallel to the x-axis and thus in the melt flow direction, the maximum birefringence (yz-plane) is determined as 5 × 10^−4^ at the sample surface. The birefringence then decreases exponentially and saturates at values around 1.8 × 10^−4^, in a depth of 0.4 mm. In this measurement direction, the results are independent of the sample position. In contrast, when measuring the birefringence along the COC plate’s y-axis, i.e., orthogonal to the melt flow direction (xz-plane), the maximum birefringence can increase significantly and is also position-dependent. At position A, the maximum birefringence as well as its depth profile are comparable to the situation parallel to the yz-plane. However, at positions B and C, maximum birefringence values of up to 8.4 × 10^−4^ were determined. Again, the values were compared to the surface birefringence obtained by means of the prism coupler. The results are summarized in Table 2.

In general, the surface birefringence values determined with both experimental setups are comparable. While the surface birefringence is constant in the yz-plane, the determined values in the xz-plane are position-dependent, whereas position A exhibits the lowest and C the largest birefringence value. It is worthwhile to note, however, that the surface birefringence determined with the prism coupler is approximately 10% larger than the values determined with the polarimeter.

This is attributed to the fact that the imaging-based evaluation technique suffers from inaccuracies near the edges of samples with finite expansion in the measurement direction. This is especially true at large magnifications since they come with a limited depth of focus. It is consequently assumed that the polarimeter underestimates the surface birefringence of the examined COC specimens.

Overall, the determined results correlate well with the known formation of near-surface birefringent regions when amorphous thermoplastics are injection-molded [24]. As birefringence directly correlates with the local degree of molecular orientation [23,25], the results allow interpretation of the respective preferred polymer chain orientation direction within the injection-molded COC plates. Overall, a high degree of molecular orientation is located in the near-surface regions close to the injection gate, while a linear decrease in molecular orientation with increasing distances from the gate is determined. At the center of the COC plate, the birefringence and thus the molecular orientation is significantly reduced and constant throughout the whole specimen. The fact that the maximum optical birefringence observed in the xz-plane is significantly higher than in the yz-plane leads to the conclusion that the preferred polymer chain orientation direction is parallel to the melt flow direction in these regions.

### 3.2. Impact on Mechanical Properties

**Figure 8:**
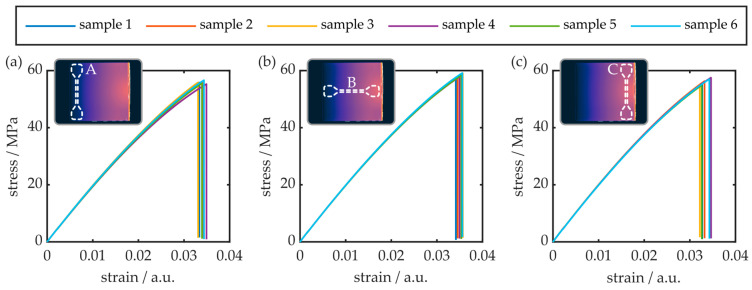
Stress–strain analysis results in different regions of the injection-molded COC plate. The samples in (**a**–**c**) represent the tensile behavior in y-direction of the least birefringent region, the tensile behavior in x-direction of the COC plate’s central region and the tensile behavior in y-direction of the most birefringent region, respectively. The insets outline the respective sample position in the COC plate’s xy-plane.

Multiple strain gauges from different regions of the birefringent COC plate were manufactured and their stress–strain behavior was examined. Overall, 18 specimens, i.e., 6 samples per position, were evaluated. The results as well as the respective sample positioning is shown in Figure 8.

Generally, all examined COC samples exhibited brittle behavior. This means that rupture occurs suddenly, without prior indication of plastic deformation. The average values for the respective tensile modulus, tensile strength as well as elongation at break were determined from the stress–strain analysis data and are summarized in Table 3.

While the tensile strength is comparable, the determined tensile modulus as well as the elongation at break values differ from previous data and supplier specifications [34,35]. This is attributed to the fact that the employed sample geometry is significantly different. However, while the impact of the sample positioning and orientation of the tensile properties is not pronounced, a comparison of the obtained data still indicates a non-negligible impact of the local molecular orientation on the COC plates’ behavior. Especially the sample from position C, which is oriented orthogonally to the preferred polymer chain orientation direction, exhibits the largest tensile modulus and the smallest elongation at break. Furthermore, this specimen group exhibits increased standard deviation values for all parameters. This infers that a high degree of molecular orientation has a negative impact on the mechanical reliability of injection-molded COC. However, the results first and foremost demonstrate that a quantitative analysis and assessment of the birefringence in injection-molded COC plates by means of classical stress–strain analysis is difficult.

**Figure 9:**
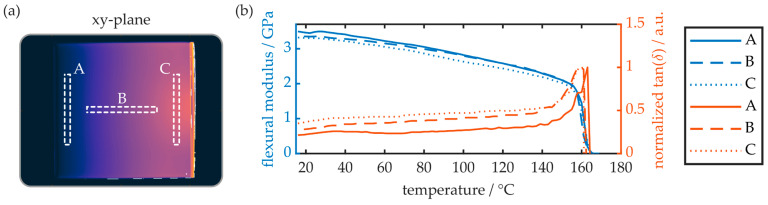
(**a**) Positioning of the samples examined via DMTA analysis. (**b**) DMTA analysis results of COC samples from different locations and thus birefringent properties. Each data set is the average of two samples.

The flexural properties of the COC plates as well as the glass transition temperature were determined via DMTA analysis. Therefore, appropriate samples were cut from the same regions of the plate, as outlined in Figure 9a. The respective results, i.e., flexural modulus as well as normalized tan(δ), in a temperature range from 18 °C to 180 °C, are shown in Figure 9b.

The overall behavior of the flexural modulus is equal for all three sample locations. At room temperature (20 °C) and up to 30 °C, the flexural modulus is at a maximum, with values of 3461 MPa, 3351 MPa and 3307 MPa for the regions A, B and C, respectively. With increasing temperatures of up to 150 °C, the flexural modus decreases linearly. Afterwards, based on the sudden and drastic reduction of the flexural modulus, the glass transition range is reached. It is worthwhile to note that no rubbery plateau was observed, which means that the injection-molded COC does not exhibit a rubbery state. Instead, all specimens directly translate from the glassy to the viscous state. The glass transition temperature is provided by the location of the tan(δ) maximum, which is found at 163 °C for the samples originating from region A. For specimens B and C, however, the determined glass transition temperature reduces to 159 °C.

While the observed flexural modulus values are in good agreement with the respective literature and the specifications of the wrought material [7,36], the glass transition temperature values are noticeably reduced in comparison with earlier studies [37]. A possible explanation for this is the impact of deviating injection-molding parameters. Furthermore, the results indicate that increased molecular orientation has a negative impact on the glass transition temperature of injection-molded COCs. However, as with stress–strain analysis, the impact of the COC plate’s local birefringence on the mechanical parameters determined via DMTA are not well pronounced. DMTA as well as static stress–strain analysis are thus suboptimal methods to assess the impact of a COC substrate’s birefringence on integrated photonic structures.

### 3.3. Impact on the Properties of Near-Surface Integrated Waveguides and Bragg Gratings

**Figure 10:**
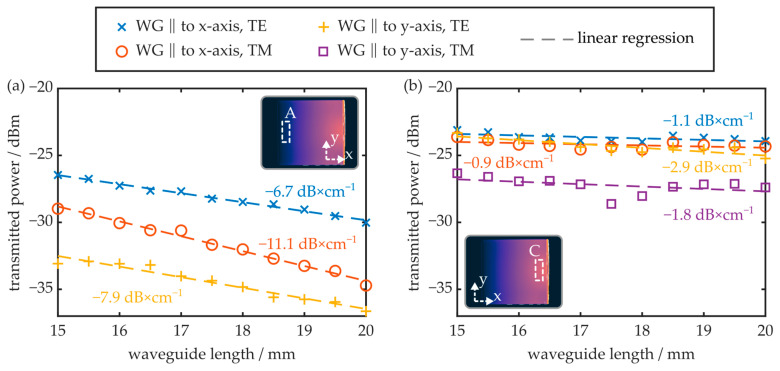
Transmitted power of TE and TM mode as a function of the waveguide length for samples located at (**a**) region A or (**b**) region C. The waveguides are either oriented parallel to the x- or the y-axis (see insets).

Multiple near-surface waveguides were fabricated in different regions of the injection-molded COC. Since they exhibited the most different behavior with respect to their inherent birefringence, the upcoming experiments focus on regions A (opposite to the injection gate) and C (close to the injection gate). In both regions, waveguides oriented parallel to the plate’s x- and y-axis were generated, and the polarization-dependent transmitted power, at wavelengths around 1550 nm, was determined as a function of the waveguide length via the cut-back method. The results are summarized in Figure 10.

It was found that the transmission signals of all waveguides fabricated in region C are significantly larger than those of the waveguides fabricated in region A. Furthermore, the optical attenuation achieved in region C is significantly smaller. Here, the TE mode of the waveguide oriented parallelly to the x-axis exhibits an attenuation of −1.1 dB × cm^−1^. In region A, however, the minimum attenuation amounts to −6.7 dB × cm^−1^ for the TE mode of the waveguide oriented parallel to the x-axis. Furthermore, in this region, no optical radiation is discernable if the waveguide is oriented parallel to the y-axis and its TM mode is excited. The results also show that waveguides oriented parallel to the x-axis, and thus parallel to the preferred orientation of the polymer chains, exhibit a reduced attenuation in comparison to waveguides oriented parallel to the y-axis. In terms of optical attenuation, it is thus preferable to fabricate near-surface waveguides in the birefringent zone of the COC plate and parallel to the melt flow direction. On the other hand, waveguides generated in region A, and parallel to the COC plate’s y-axis, have the potential to serve as modal filters, since they do not transmit the TM mode while the TE mode is guided with reasonable attenuation.

**Figure 11:**
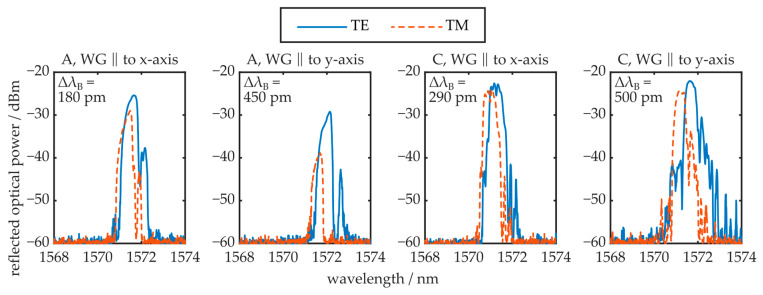
TE and TM polarization mode reflection signals of Bragg gratings from different regions (A and C, see Figure 10) and with different orientations (WG ‖ to x-axis and WG ‖ to y-axis). Furthermore, the Bragg wavelength difference Δλ_B_ of both polarizations is stated.

The local birefringence of the injection-molded COC plate also influences the BG’s reflection signal, as demonstrated in Figure 11. Again, waveguides and BGs located in regions A and C, oriented either parallel to the x- or the y-axis, are examined.

The results show that the obtained reflection from BGs located in region C is larger compared to the reflected optical power received by photonic structures fabricated in region A. This observation correlates well with the respective transmission signals and optical attenuation values determined for both regions. Furthermore, the difference in reflection peak amplitude of the polarization modes can also be unambiguously attributed to the determined, polarization-dependent loss properties of the respective waveguide orientations. In contrast to the transmission signal configuration, a residual TM mode reflection is determined from the photonic structures oriented parallelly to the y-axis, located in region A. However, the TM mode’s reflection peak amplitude is reduced by 10 dB in comparison to the TE mode’s reflection signal. It is assumed that the significantly shorter propagation distance in the reflective configuration enables detection of the residual TM mode signal. Qualitatively, the observed Bragg wavelength difference of the polarization modes follows the local near-surface birefringence behavior of the COC plate, i.e., increased birefringence values lead to a more pronounced peak splitting. However, the quantitative values do not correlate with the determined local birefringence. According to Equation (10), the polarization-dependent peak splitting yields values of half the near-surface birefringence in most cases. This behavior, as well as the observed deviations in optical waveguide attenuation (see Figure 10), are both correlated with the fact that the chemical material modification processes triggered by UV-light irradiation and thermal treatment, which lead to the formation of photonic structures, are based on reorientation and reconfiguration of the macromolecules, i.e., polymer chain scission [38,39]. Consequently, the birefringence in the irradiated and thus modified area can differ from the pristine COC plate. It is worthwhile to note that the TE and TM peak Bragg wavelength difference of the BG oriented parallel to the y-axis in region A does not follow this behavior. However, since the TM mode is not well supported in this configuration, the peak splitting is not caused by the near-surface birefringence alone. The weak waveguiding of the TM mode possibly leads to penetration into the pristine and unmodified COC material, which exhibits a significantly reduced refractive index. It is worthwhile to note that, in practice, most Bragg grating devices are evaluated with unpolarized radiation. In this case, the reflection spectrum comprises the superposition of the TE and TM reflection peaks. In cases where the spectral separation of the polarization mode reflection peaks is sufficiently pronounced, this leads to visible peak splitting in the unpolarized reflection signal, an unwanted property in most cases. With this in mind, fabrication of BGs in region A of the COC plate is advantageous, since these structures show sufficient reflection peak amplitude but negligible peak splitting effects.

**Figure 12:**
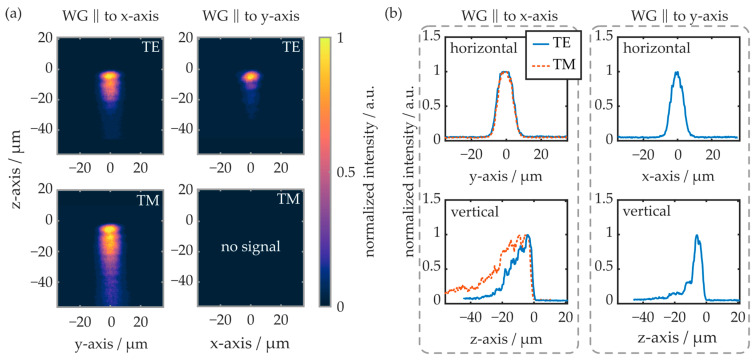
(**a**) False-color representation of the near-field intensity distribution within waveguides located in region A of the COC plate. (**b**) Line plots of the horizontal and vertical cross-sectional intensities. TE and TM modes of the differently oriented waveguides are excited and evaluated separately. On the z-axis (vertical), the origin represents the substrate surface.

The polarization-dependent modal intensity distribution was examined by means of the near-field setup. First, the waveguides located in region A of the COC plate are evaluated, as shown in Figure 12.

Again, like in previous experiments, the TM mode is not sufficiently supported by the waveguide oriented parallel to the y-axis. Thus, there is no evaluable near-field signal. In the horizontal plane, the modal shape is comparable in all cases, independent of the waveguide’s orientation and the polarization mode. In the vertical plane, however, the waveguide orientation and the excited polarization influence the near-field intensity distribution significantly. If the waveguide is oriented parallelly to the y-axis, the TE mode reaches about 20 µm into the substrate (e^−2^-value). If the waveguide is oriented parallelly to the x-axis, the TE mode penetrates 26 µm deep into the substrate, while the TM mode’s edge is located more than 55 µm underneath the substrate surface.

**Figure 13:**
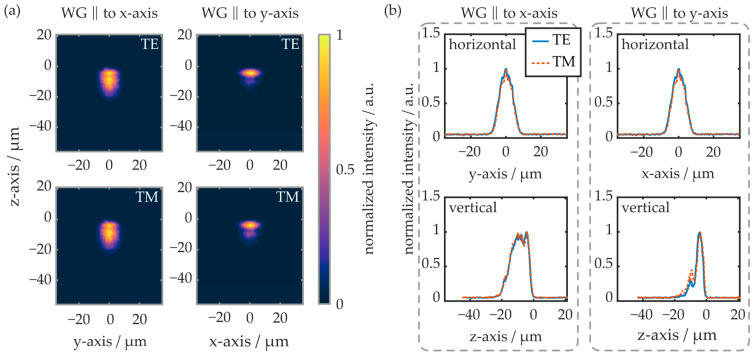
(**a**) False-color representation of the near-field intensity distribution within waveguides located in region C of the COC plate. (**b**) Line plots of the horizontal and vertical cross-sectional intensities. TE and TM modes of the differently oriented waveguides are excited and evaluated separately. On the z-axis (vertical), the origin represents the substrate surface.

The near-field evaluation results of similarly oriented waveguides from region C of the COC plate are depicted in Figure 13. In this region, the waveguide parallel to the y-axis supports both polarization modes. Overall, the radiation is spatially more tightly confined compared to waveguides fabricated in region A of the plate. While, on the horizontal cross-section, the intensity distribution is comparable to the waveguides generated in region A, the radiation propagates closer to the substrate surface on the vertical cross-section. For waveguides oriented parallelly to the y-axis, the radiation penetrates up to 13 µm into the substrate, while it reaches up to 20 µm into the COC in waveguides oriented parallelly to the x-axis. Overall, the results of the near-field evaluation correlate well with the determined transmission signal and waveguide attenuation properties. First, the reduced spatial extension of the waveguide modes leads to an increased modal overlap between the butt-coupled fiber-optic pigtail and the integrated waveguide.

This, in turn, yields significantly reduced coupling losses and thus an increase in transmission signal. This thesis is furthermore supported by the observed Bragg reflection peak amplitudes, which are also larger for BGs located in region C of the COC plate. Second, the increased modal confinement of waveguides fabricated in region C indicates stronger waveguiding, which is in good agreement with the determined optical attenuation values. As all waveguides are fabricated with the same parameters, this underlines that irradiation of the COC plate regions with pronounced birefringence leads to favorable waveguiding conditions.

### 3.4. Buried Waveguides and Bragg Gratings

**Figure 14:**
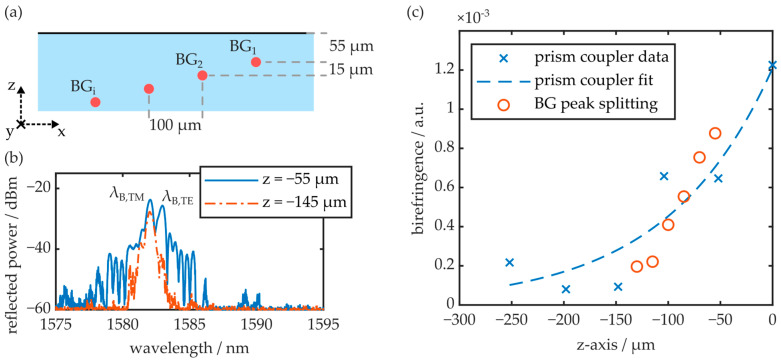
(**a**) Positioning of the fs laser-generated waveguides and BGs within the COC substrate. (**b**) Exemplary reflection peaks of BGs at depths of 55 µm and 145 µm underneath the substrate surface. (**c**) Comparison of the birefringence depth profile determined with the prism coupler and the birefringence values calculated from the peak splitting of the integrated BGs.

Multiple waveguides, oriented parallelly to a COC plate’s y-axis, were fabricated in region C of an injection-molded plate by means of the femtosecond laser-based direct writing approach. The waveguides were generated at different depths underneath the substrate surface, whereas the uppermost photonic structure was located at a depth of 55 µm. The other waveguides were fabricated at increasing depths, with a step width of 15 µm. All waveguides were laterally offset by 100 µm in x-direction to ensure that they did not interfere with each other and the structures were not irradiated repeatedly during fabrication. A schematic of the layout is given in Figure 14a.

All waveguides were equipped with Bragg gratings. Figure 14b depicts the reflection signal of two photonic structures fabricated at depths of 55 µm and 145 µm, respectively. The waveguides are excited with unpolarized light. Thus, the reflection spectrum comprises the superimposed signal of the TE and the TM reflection peaks. While the BG close to the surface exhibits significant peak splitting, the BG buried deeper in the substrate comprises a single reflection peak, as it is located in the less birefringent volume of the substrate. The birefringence, calculated according to Equation (10) from the determined peak splitting, is visualized as a function of the BG’s z-axis position in Figure 14c. The graph furthermore comprises the substrate’s birefringence depth profile acquired via the prism coupler. Therefore, the substrate thickness is reduced layer by layer with the micro mill and then polished back to optical quality in between measurements. The birefringence depth profile agrees well with the birefringence data acquired via polarimetry (see Figure 7).

Furthermore, the data shows an unambiguous correlation between the peak splitting and the birefringence depth profile. Finally, the experiment demonstrates that unwanted peak splitting effects, exhibited by near-surface BGs fabricated in injection-molded COC plates, can be completely omitted by positioning the photonic structures deeper in the substrate volume.

### 3.5. Guidelines for the Fabrication of Photonic Structures in Injection-Molded Cyclic Olefin Copolymer Substrates

Based on the observations discussed in this work, it is possible to outline some general guidelines for the fabrication of photonic structures in injection-molded COCs. While it is theoretically possible to influence the degree of molecular orientation in the workpieces by means of adapting the injection-molding parameters, e.g., process and mold temperature, injection and back pressure as well as mold geometry [40], it is challenging to avoid these effects completely. Nevertheless, injection molding is the preferred choice to manufacture low-cost and large-quantity polymer-based products, a prerequisite for commercial success in attractive markets, such as medical platforms and sensing applications [21,22]. Therefore, as the behavior of near-surface integrated waveguides and BGs strongly depends on their location and orientation, the inherent birefringence of the injection-molded plates needs to be adequately quantified.

According to this study, mechanical analysis is insufficient to appropriately detect and analyze the anisotropic properties of the workpieces. Instead, the usage of optical measurement techniques in the form of a prism coupler or a polarimeter is mandatory. Analysis of the birefringence in the plane parallel to the melt flow (xy-plane) is sufficient to derive some basic design decisions. For example, if the intended application requires low-loss waveguides, fabrication of the photonic structures in the more birefringent area (region C, near the injection gate) is recommended. If, on the other hand, peak splitting or double peaks in the reflection spectrum of a BG-based device are to be avoided, the photonic structures need to be located in the less birefringent region of the injection-molded plate (region A). However, in order to accurately predict the behavior of a near-surface integrated photonic structure, three-dimensional analysis of the injection-molded plate’s birefringence is necessary. This is the only way to quantify the inhomogeneous molecular orientation distribution in the COC substrates. Alternatively, femtosecond laser direct writing processes can omit birefringence effects completely, as they allow the fabrication of photonic structures deeper within the volume of the substrate. Consequently, this fabrication method is preferred if the target application does not necessitate near-surface structures, as in the case of devices that interact with sensitization coatings [12,13].

## 4. Conclusions

In summary, this article provides sophisticated insight into the birefringence of injection-molded COC plates and its impact on integrated photonic structures. First, the birefringence distribution in all three dimensions was quantified by means of polarimetry in combination with a prism coupler setup. It was found that in the xy-plane, the COC plates exhibit maximum birefringence near the injection gate. With a larger distance from the gate, the birefringence reduces linearly and reaches zero at the opposite end of the plate. In the xz- and the yz-plane, a significant birefringence was observed near the surface of the COC material. Based on the measurement location, the maximum birefringence at the surface was determined as 10 × 10^−4^, while it reduced exponentially to values below 2 × 10^−4^ towards the center of the plate. Second, mechanical analyses, including stress–strain and DMTA experiments of the birefringent COC material, led to the result that it is difficult to discern and evaluate the birefringence with these approaches. While increased standard deviations during stress–strain measurements and reduced glass transition temperatures during DMTA analysis can serve as indicators for the presence of material anisotropies, the meaningfulness of these methods is insufficient.

However, a distinct impact of the birefringence was correlated with the behavior of near-surface waveguides and Bragg gratings in the outline of this study. It was found that the birefringence unambiguously influences the transmission and attenuation properties. On the one hand, the experiments show that near-surface waveguides with an attenuation around 1 dB cm^−1^, for both of the waveguide’s polarization modes, can be manufactured in the birefringent region near the injection gate (region C). On the other hand, it is possible to fabricate waveguides on the opposite side of the plate (region A) that do not support the TM mode, while the TE mode is guided with an attenuation of −7.9 dB cm^−1^. It is thus feasible to realize polarization mode filters in this region of the injection-molded plate. Moreover, the impact of the birefringence of near-surface Bragg gratings was evaluated. The study shows that the birefringence of the injection-molded substrate results in a distinct reflection peak for each of the polarization modes. This leads to unwanted peak splitting if the BG structure is evaluated by means of unpolarized radiation. Thus, it is advantageous to fabricate near-surface BGs in areas with low birefringence, i.e., in region A of the COC plate. It is also possible to omit the birefringence impact by positioning the photonic structures deeper in the substrate volume. Femtosecond laser-fabricated waveguides with integrated BGs do not exhibit peak splitting when they are fabricated at least 145 µm underneath the substrate surface.

In conclusion, this contribution underlines the importance of considering the birefringence of injection-molded substrates for the fabrication of near-surface integrated photonic devices. Besides quantifying the birefringence at the position of the photonic structure, it is also important to consider the waveguide orientation as well as the intended application. It also points out that adequate process stability and control are necessary to obtain repeatable results when fabricating near-surface photonic structures within injection-molded COC plates. This study therefore provides design guidelines for the fabrication of integrated photonic structures within injection-molded cyclic olefin copolymer substrates.

## Figures and Tables

**Table 1:** Overview of the employed process parameters for the femtosecond laser-based fabrication of buried waveguides and Bragg gratings in injection-molded COC.

Parameter	Waveguide	Bragg Grating
wavelength/nm	514	
pulse energy/nJ	330	
pulse duration/fs	450	
repetition rate/kHz	25	0.5
translation speed/mm × s^−1^	15	0.52
focal cross-section/µm	15 × 1.5 ^1^	

^1^ The major axis of the elliptical cross-section is oriented parallelly to the x-axis.

**Table 2:** Surface birefringence at positions A, B and C, determined via prism coupler.

Position	Surface Birefringence/a.u.	
	xz-Plane	yz-Plane
A	6 × 10^−4^	4 × 10^−4^
B	7 × 10^−4^	5 × 10^−4^
C	10 × 10^−4^	4 × 10^−4^

**Table 3:** Stress–strain analysis parameters of different positions and sample orientations within injection-molded COC plates.

Position	Average Value (1σ Standard Deviation)		
	Tensile Modulus/MPa	Tensile Strength/MPa	Elongation at Break/%
A	1991 (24)	56 (0.5)	3.4 (7 × 10^−2^)
B	2011 (5)	58 (0.9)	3.5 (6 × 10^−2^)
C	2040 (32)	56 (1.1)	3.3 (10 × 10^−2^)

## Data Availability

Data underlying the results presented in this paper are not publicly available at this time but may be obtained from the authors upon reasonable request.
